# Updated search for long-lived particles decaying to jet pairs

**DOI:** 10.1140/epjc/s10052-017-5178-x

**Published:** 2017-11-29

**Authors:** R. Aaij, B. Adeva, M. Adinolfi, Z. Ajaltouni, S. Akar, J. Albrecht, F. Alessio, M. Alexander, S. Ali, G. Alkhazov, P. Alvarez Cartelle, A. A. Alves, S. Amato, S. Amerio, Y. Amhis, L. An, L. Anderlini, G. Andreassi, M. Andreotti, J. E. Andrews, R. B. Appleby, F. Archilli, P. d’Argent, J. Arnau Romeu, A. Artamonov, M. Artuso, E. Aslanides, G. Auriemma, M. Baalouch, I. Babuschkin, S. Bachmann, J. J. Back, A. Badalov, C. Baesso, S. Baker, V. Balagura, W. Baldini, A. Baranov, R. J. Barlow, C. Barschel, S. Barsuk, W. Barter, F. Baryshnikov, M. Baszczyk, V. Batozskaya, V. Battista, A. Bay, L. Beaucourt, J. Beddow, F. Bedeschi, I. Bediaga, A. Beiter, L. J. Bel, V. Bellee, N. Belloli, K. Belous, I. Belyaev, E. Ben-Haim, G. Bencivenni, S. Benson, S. Beranek, A. Berezhnoy, R. Bernet, A. Bertolin, C. Betancourt, F. Betti, M.-O. Bettler, M. van Beuzekom, Ia. Bezshyiko, S. Bifani, P. Billoir, T. Bird, A. Birnkraut, A. Bitadze, A. Bizzeti, T. Blake, F. Blanc, J. Blouw, S. Blusk, V. Bocci, T. Boettcher, A. Bondar, N. Bondar, W. Bonivento, I. Bordyuzhin, A. Borgheresi, S. Borghi, M. Borisyak, M. Borsato, F. Bossu, M. Boubdir, T. J. V. Bowcock, E. Bowen, C. Bozzi, S. Braun, M. Britsch, T. Britton, J. Brodzicka, E. Buchanan, C. Burr, A. Bursche, J. Buytaert, S. Cadeddu, R. Calabrese, M. Calvi, M. Calvo Gomez, A. Camboni, P. Campana, D. H. Campora Perez, L. Capriotti, A. Carbone, G. Carboni, R. Cardinale, A. Cardini, P. Carniti, L. Carson, K. Carvalho Akiba, G. Casse, L. Cassina, L. Castillo Garcia, M. Cattaneo, G. Cavallero, R. Cenci, D. Chamont, M. Charles, Ph. Charpentier, G. Chatzikonstantinidis, M. Chefdeville, S. Chen, S. F. Cheung, V. Chobanova, M. Chrzaszcz, X. Cid Vidal, G. Ciezarek, P. E. L. Clarke, M. Clemencic, H. V. Cliff, J. Closier, V. Coco, J. Cogan, E. Cogneras, V. Cogoni, L. Cojocariu, P. Collins, A. Comerma-Montells, A. Contu, A. Cook, G. Coombs, S. Coquereau, G. Corti, M. Corvo, C. M. Costa Sobral, B. Couturier, G. A. Cowan, D. C. Craik, A. Crocombe, M. Cruz Torres, S. Cunliffe, R. Currie, C. D’Ambrosio, F. Da Cunha Marinho, E. Dall’Occo, J. Dalseno, P. N. Y. David, A. Davis, K. De Bruyn, S. De Capua, M. De Cian, J. M. De Miranda, L. De Paula, M. De Serio, P. De Simone, C. T. Dean, D. Decamp, M. Deckenhoff, L. Del Buono, M. Demmer, A. Dendek, D. Derkach, O. Deschamps, F. Dettori, B. Dey, A. Di Canto, H. Dijkstra, F. Dordei, M. Dorigo, A. Dosil Suárez, A. Dovbnya, K. Dreimanis, L. Dufour, G. Dujany, K. Dungs, P. Durante, R. Dzhelyadin, A. Dziurda, A. Dzyuba, N. Déléage, S. Easo, M. Ebert, U. Egede, V. Egorychev, S. Eidelman, S. Eisenhardt, U. Eitschberger, R. Ekelhof, L. Eklund, S. Ely, S. Esen, H. M. Evans, T. Evans, A. Falabella, N. Farley, S. Farry, R. Fay, D. Fazzini, D. Ferguson, G. Fernandez, A. Fernandez Prieto, F. Ferrari, F. Ferreira Rodrigues, M. Ferro-Luzzi, S. Filippov, R. A. Fini, M. Fiore, M. Fiorini, M. Firlej, C. Fitzpatrick, T. Fiutowski, F. Fleuret, K. Fohl, M. Fontana, F. Fontanelli, D. C. Forshaw, R. Forty, V. Franco Lima, M. Frank, C. Frei, J. Fu, W. Funk, E. Furfaro, C. Färber, A. Gallas Torreira, D. Galli, S. Gallorini, S. Gambetta, M. Gandelman, P. Gandini, Y. Gao, L. M. Garcia Martin, J. García Pardiñas, J. Garra Tico, L. Garrido, P. J. Garsed, D. Gascon, C. Gaspar, L. Gavardi, G. Gazzoni, D. Gerick, E. Gersabeck, M. Gersabeck, T. Gershon, Ph. Ghez, S. Gianì, V. Gibson, O. G. Girard, L. Giubega, K. Gizdov, V. V. Gligorov, D. Golubkov, A. Golutvin, A. Gomes, I. V. Gorelov, C. Gotti, E. Govorkova, R. Graciani Diaz, L. A. Granado Cardoso, E. Graugés, E. Graverini, G. Graziani, A. Grecu, R. Greim, P. Griffith, L. Grillo, B. R. Gruberg Cazon, O. Grünberg, E. Gushchin, Yu. Guz, T. Gys, C. Göbel, T. Hadavizadeh, C. Hadjivasiliou, G. Haefeli, C. Haen, S. C. Haines, B. Hamilton, X. Han, S. Hansmann-Menzemer, N. Harnew, S. T. Harnew, J. Harrison, M. Hatch, J. He, T. Head, A. Heister, K. Hennessy, P. Henrard, L. Henry, E. van Herwijnen, M. Heß, A. Hicheur, D. Hill, C. Hombach, P. H. Hopchev, W. Hulsbergen, T. Humair, M. Hushchyn, D. Hutchcroft, M. Idzik, P. Ilten, R. Jacobsson, A. Jaeger, J. Jalocha, E. Jans, A. Jawahery, F. Jiang, M. John, D. Johnson, C. R. Jones, C. Joram, B. Jost, N. Jurik, S. Kandybei, M. Karacson, J. M. Kariuki, S. Karodia, M. Kecke, M. Kelsey, M. Kenzie, T. Ketel, E. Khairullin, B. Khanji, C. Khurewathanakul, T. Kirn, S. Klaver, K. Klimaszewski, T. Klimkovich, S. Koliiev, M. Kolpin, I. Komarov, P. Koppenburg, A. Kosmyntseva, M. Kozeiha, L. Kravchuk, K. Kreplin, M. Kreps, P. Krokovny, F. Kruse, W. Krzemien, W. Kucewicz, M. Kucharczyk, V. Kudryavtsev, A. K. Kuonen, K. Kurek, T. Kvaratskheliya, D. Lacarrere, G. Lafferty, A. Lai, G. Lanfranchi, C. Langenbruch, T. Latham, C. Lazzeroni, R. Le Gac, J. van Leerdam, A. Leflat, J. Lefrançois, R. Lefèvre, F. Lemaitre, E. Lemos Cid, O. Leroy, T. Lesiak, B. Leverington, T. Li, Y. Li, T. Likhomanenko, R. Lindner, C. Linn, F. Lionetto, X. Liu, D. Loh, I. Longstaff, J. H. Lopes, D. Lucchesi, M. Lucio Martinez, H. Luo, A. Lupato, E. Luppi, O. Lupton, A. Lusiani, X. Lyu, F. Machefert, F. Maciuc, O. Maev, K. Maguire, S. Malde, A. Malinin, T. Maltsev, G. Manca, G. Mancinelli, P. Manning, J. Maratas, J. F. Marchand, U. Marconi, C. Marin Benito, M. Marinangeli, P. Marino, J. Marks, G. Martellotti, M. Martin, M. Martinelli, D. Martinez Santos, F. Martinez Vidal, D. Martins Tostes, L. M. Massacrier, A. Massafferri, R. Matev, A. Mathad, Z. Mathe, C. Matteuzzi, A. Mauri, E. Maurice, B. Maurin, A. Mazurov, M. McCann, A. McNab, R. McNulty, B. Meadows, F. Meier, M. Meissner, D. Melnychuk, M. Merk, A. Merli, E. Michielin, D. A. Milanes, M.-N. Minard, D. S. Mitzel, A. Mogini, J. Molina Rodriguez, I. A. Monroy, S. Monteil, M. Morandin, P. Morawski, A. Mordà, M. J. Morello, O. Morgunova, J. Moron, A. B. Morris, R. Mountain, F. Muheim, M. Mulder, M. Mussini, D. Müller, J. Müller, K. Müller, V. Müller, P. Naik, T. Nakada, R. Nandakumar, A. Nandi, I. Nasteva, M. Needham, N. Neri, S. Neubert, N. Neufeld, M. Neuner, T. D. Nguyen, C. Nguyen-Mau, S. Nieswand, R. Niet, N. Nikitin, T. Nikodem, A. Nogay, A. Novoselov, D. P. O’Hanlon, A. Oblakowska-Mucha, V. Obraztsov, S. Ogilvy, R. Oldeman, C. J. G. Onderwater, J. M. Otalora Goicochea, A. Otto, P. Owen, A. Oyanguren, P. R. Pais, A. Palano, M. Palutan, A. Papanestis, M. Pappagallo, L. L. Pappalardo, W. Parker, C. Parkes, G. Passaleva, A. Pastore, G. D. Patel, M. Patel, C. Patrignani, A. Pearce, A. Pellegrino, G. Penso, M. Pepe Altarelli, S. Perazzini, P. Perret, L. Pescatore, K. Petridis, A. Petrolini, A. Petrov, M. Petruzzo, E. Picatoste Olloqui, B. Pietrzyk, M. Pikies, D. Pinci, A. Pistone, A. Piucci, V. Placinta, S. Playfer, M. Plo Casasus, T. Poikela, F. Polci, A. Poluektov, I. Polyakov, E. Polycarpo, G. J. Pomery, S. Ponce, A. Popov, D. Popov, B. Popovici, S. Poslavskii, C. Potterat, E. Price, J. D. Price, J. Prisciandaro, A. Pritchard, C. Prouve, V. Pugatch, A. Puig Navarro, G. Punzi, W. Qian, R. Quagliani, B. Rachwal, J. H. Rademacker, M. Rama, M. Ramos Pernas, M. S. Rangel, I. Raniuk, F. Ratnikov, G. Raven, F. Redi, S. Reichert, A. C. dos Reis, C. Remon Alepuz, V. Renaudin, S. Ricciardi, S. Richards, M. Rihl, K. Rinnert, V. Rives Molina, P. Robbe, A. B. Rodrigues, E. Rodrigues, J. A. Rodriguez Lopez, P. Rodriguez Perez, A. Rogozhnikov, S. Roiser, A. Rollings, V. Romanovskiy, A. Romero Vidal, J. W. Ronayne, M. Rotondo, M. S. Rudolph, T. Ruf, P. Ruiz Valls, J. J. Saborido Silva, E. Sadykhov, N. Sagidova, B. Saitta, V. Salustino Guimaraes, D. Sanchez Gonzalo, C. Sanchez Mayordomo, B. Sanmartin Sedes, R. Santacesaria, C. Santamarina Rios, M. Santimaria, E. Santovetti, A. Sarti, C. Satriano, A. Satta, D. M. Saunders, D. Savrina, S. Schael, M. Schellenberg, M. Schiller, H. Schindler, M. Schlupp, M. Schmelling, T. Schmelzer, B. Schmidt, O. Schneider, A. Schopper, H. F. Schreiner, K. Schubert, M. Schubiger, M.-H. Schune, R. Schwemmer, B. Sciascia, A. Sciubba, A. Semennikov, A. Sergi, N. Serra, J. Serrano, L. Sestini, P. Seyfert, M. Shapkin, I. Shapoval, Y. Shcheglov, T. Shears, L. Shekhtman, V. Shevchenko, B. G. Siddi, R. Silva Coutinho, L. Silva de Oliveira, G. Simi, S. Simone, M. Sirendi, N. Skidmore, T. Skwarnicki, E. Smith, I. T. Smith, J. Smith, M. Smith, l. Soares Lavra, M. D. Sokoloff, F. J. P. Soler, B. Souza De Paula, B. Spaan, P. Spradlin, S. Sridharan, F. Stagni, M. Stahl, S. Stahl, P. Stefko, S. Stefkova, O. Steinkamp, S. Stemmle, O. Stenyakin, H. Stevens, S. Stevenson, S. Stoica, S. Stone, B. Storaci, S. Stracka, M. E. Stramaglia, M. Straticiuc, U. Straumann, L. Sun, W. Sutcliffe, K. Swientek, V. Syropoulos, M. Szczekowski, T. Szumlak, S. T’Jampens, A. Tayduganov, T. Tekampe, G. Tellarini, F. Teubert, E. Thomas, J. van Tilburg, M. J. Tilley, V. Tisserand, M. Tobin, S. Tolk, L. Tomassetti, D. Tonelli, S. Topp-Joergensen, F. Toriello, E. Tournefier, S. Tourneur, K. Trabelsi, M. Traill, M. T. Tran, M. Tresch, A. Trisovic, A. Tsaregorodtsev, P. Tsopelas, A. Tully, N. Tuning, A. Ukleja, A. Ustyuzhanin, U. Uwer, C. Vacca, V. Vagnoni, A. Valassi, S. Valat, G. Valenti, R. Vazquez Gomez, P. Vazquez Regueiro, S. Vecchi, M. van Veghel, J. J. Velthuis, M. Veltri, G. Veneziano, A. Venkateswaran, M. Vernet, M. Vesterinen, J. V. Viana Barbosa, B. Viaud, D. Vieira, M. Vieites Diaz, H. Viemann, X. Vilasis-Cardona, M. Vitti, V. Volkov, A. Vollhardt, B. Voneki, A. Vorobyev, V. Vorobyev, C. Voß, J. A. de Vries, C. Vázquez Sierra, R. Waldi, C. Wallace, R. Wallace, J. Walsh, J. Wang, D. R. Ward, H. M. Wark, N. K. Watson, D. Websdale, A. Weiden, M. Whitehead, J. Wicht, G. Wilkinson, M. Wilkinson, M. Williams, M. P. Williams, M. Williams, T. Williams, F. F. Wilson, J. Wimberley, M. A. Winn, J. Wishahi, W. Wislicki, M. Witek, G. Wormser, S. A. Wotton, K. Wraight, K. Wyllie, Y. Xie, Z. Xu, Z. Yang, Y. Yao, H. Yin, J. Yu, X. Yuan, O. Yushchenko, K. A. Zarebski, M. Zavertyaev, L. Zhang, Y. Zhang, A. Zhelezov, Y. Zheng, X. Zhu, V. Zhukov, S. Zucchelli, LHCb Collaboration

**Affiliations:** 1 Centro Brasileiro de Pesquisas Físicas (CBPF), Rio de Janeiro, Brazil; 2 Universidade Federal do Rio de Janeiro (UFRJ), Rio de Janeiro, Brazil; 3 Center for High Energy Physics, Tsinghua University, Beijing, China; 4 LAPP, Université Savoie Mont-Blanc, CNRS/IN2P3, Annecy-Le-Vieux, France; 5 Clermont Université, Université Blaise Pascal, CNRS/IN2P3, LPC, Clermont-Ferrand, France; 6 CPPM, Aix-Marseille Université, CNRS/IN2P3, Marseille, France; 7 LAL, Université Paris-Sud, CNRS/IN2P3, Orsay, France; 8 LPNHE, Université Pierre et Marie Curie, Université Paris Diderot, CNRS/IN2P3, Paris, France; 9 I. Physikalisches Institut, RWTH Aachen University, Aachen, Germany; 10 Fakultät Physik, Technische Universität Dortmund, Dortmund, Germany; 11 Max-Planck-Institut für Kernphysik (MPIK), Heidelberg, Germany; 12 Physikalisches Institut, Ruprecht-Karls-Universität Heidelberg, Heidelberg, Germany; 13 School of Physics, University College Dublin, Dublin, Ireland; 14 Sezione INFN di Bari, Bari, Italy; 15 Sezione INFN di Bologna, Bologna, Italy; 16 Sezione INFN di Cagliari, Cagliari, Italy; 17 Universita e INFN Ferrara, Ferrara, Italy; 18 Sezione INFN di Firenze, Florence, Italy; 19 Laboratori Nazionali dell’INFN di Frascati, Frascati, Italy; 20 Sezione INFN di Genova, Genoa, Italy; 21 Universita & INFN Milano Bicocca, Milan, Italy; 22 Sezione di Milano, Milan, Italy; 23 Sezione INFN di Padova, Padova, Italy; 24 Sezione INFN di Pisa, Pisa, Italy; 25 Sezione INFN di Roma Tor Vergata, Rome, Italy; 26 Sezione INFN di Roma La Sapienza, Rome, Italy; 27 Henryk Niewodniczanski Institute of Nuclear Physics Polish Academy of Sciences, Kraków, Poland; 28 Faculty of Physics and Applied Computer Science, AGH-University of Science and Technology, Kraków, Poland; 29 National Center for Nuclear Research (NCBJ), Warsaw, Poland; 30 Horia Hulubei National Institute of Physics and Nuclear Engineering, Bucharest-Magurele, Romania; 31 Petersburg Nuclear Physics Institute (PNPI), Gatchina, Russia; 32 Institute of Theoretical and Experimental Physics (ITEP), Moscow, Russia; 33 Institute of Nuclear Physics, Moscow State University (SINP MSU), Moscow, Russia; 34 Institute for Nuclear Research of the Russian Academy of Sciences (INR RAN), Moscow, Russia; 35 Yandex School of Data Analysis, Moscow, Russia; 36 Budker Institute of Nuclear Physics (SB RAS), Novosibirsk, Russia; 37 Institute for High Energy Physics (IHEP), Protvino, Russia; 38 ICCUB, Universitat de Barcelona, Barcelona, Spain; 39 Universidad de Santiago de Compostela, Santiago de Compostela, Spain; 40 European Organization for Nuclear Research (CERN), Geneva, Switzerland; 41 Institute of Physics, Ecole Polytechnique Fédérale de Lausanne (EPFL), Lausanne, Switzerland; 42 Physik-Institut, Universität Zürich, Zürich, Switzerland; 43 Nikhef National Institute for Subatomic Physics, Amsterdam, The Netherlands; 44 Nikhef National Institute for Subatomic Physics and VU University Amsterdam, Amsterdam, The Netherlands; 45 NSC Kharkiv Institute of Physics and Technology (NSC KIPT), Kharkiv, Ukraine; 46 Institute for Nuclear Research of the National Academy of Sciences (KINR), Kyiv, Ukraine; 47 University of Birmingham, Birmingham, UK; 48 H.H. Wills Physics Laboratory, University of Bristol, Bristol, UK; 49 Cavendish Laboratory, University of Cambridge, Cambridge, UK; 50 Department of Physics, University of Warwick, Coventry, UK; 51 STFC Rutherford Appleton Laboratory, Didcot, UK; 52 School of Physics and Astronomy, University of Edinburgh, Edinburgh, UK; 53 School of Physics and Astronomy, University of Glasgow, Glasgow, UK; 54 Oliver Lodge Laboratory, University of Liverpool, Liverpool, UK; 55 Imperial College London, London, UK; 56 School of Physics and Astronomy, University of Manchester, Manchester, UK; 57 Department of Physics, University of Oxford, Oxford, UK; 58 Massachusetts Institute of Technology, Cambridge, MA USA; 59 University of Cincinnati, Cincinnati, OH USA; 60 University of Maryland, College Park, MD USA; 61 Syracuse University, Syracuse, NY USA; 62 Pontifícia Universidade Católica do Rio de Janeiro (PUC-Rio), Rio de Janeiro, Brazil; 63 University of Chinese Academy of Sciences, Beijing, China; 64 School of Physics and Technology, Wuhan University, Wuhan, China; 65 Institute of Particle Physics, Central China Normal University, Wuhan, Hubei China; 66 Departamento de Fisica, Universidad Nacional de Colombia, Bogotá, Colombia; 67 Institut für Physik, Universität Rostock, Rostock, Germany; 68 National Research Centre Kurchatov Institute, Moscow, Russia; 69 Instituto de Fisica Corpuscular, Centro Mixto Universidad de Valencia-CSIC, Valencia, Spain; 70 Van Swinderen Institute, University of Groningen, Groningen, The Netherlands; 71 CERN, 1211 Geneva 23, Switzerland

## Abstract

A search is presented for long-lived particles with a mass between 25 and 50$\,\mathrm{GeV}/c^{2}$ and a lifetime between 2 and 500 ps, using proton–proton collision data corresponding to an integrated luminosity of 2.0$\,\mathrm{fb}^{-1}$, collected by the LHCb detector at centre-of-mass energies of 7 and 8 TeV. The particles are assumed to be pair-produced in the decay of a 125$\,\mathrm{GeV}/c^{2}$ Standard-Model-like Higgs boson. The experimental signature is a single long-lived particle, identified by a displaced vertex with two associated jets. No excess above background is observed and limits are set on the production cross-section as a function of the mass and lifetime of the long-lived particle.

## Introduction

Various extensions of the Standard Model (SM) feature new particles whose couplings to lighter states are sufficiently small to result in detectable lifetimes. In this paper we report on a search for such long-lived particles, which are assumed to be pair-produced in the decay of a Standard-Model-like Higgs boson, and subsequently decay into a quark–antiquark pair. Such a signature is present in models with a hidden-sector non-Abelian gauge group, where the Standard Model Higgs boson acts as a portal [1–5]. The new scalar particle represents the lightest state in the hidden sector and is called a hidden-valley pion ($\pi _{\text {v}}$) throughout this paper. Experimental constraints on the properties of the Higgs boson of mass 125$\,\mathrm{GeV}/c^{2}$ observed by the ATLAS and CMS collaborations [6, 7] still allow for branching fractions of non-SM decay modes of up to 30% [8].

Data collected with the LHCb experiment in 2011 and 2012 are used for this analysis, restricted to periods in which suitable triggers were available. The data sample analysed corresponds to 0.62$\,\mathrm{fb}^{-1}$ at a centre-of-mass energy of $\sqrt{s}={7}\,\mathrm{TeV}$ and 1.38$\,\mathrm{fb}^{-1}$ at $\sqrt{s}={8}\,\mathrm{TeV}$. In simulated events with $\pi _{\text {v}}$ pairs originating from a Higgs boson decay it is found that in most cases no more than one of the two $\pi _{\text {v}}$ decays occurs inside the LHCb acceptance. Consequently, the experimental signature is a single $\pi _{\text {v}}$ particle. The candidate is identified by its decay to two hadronic jets originating from a displaced vertex, with a transverse distance to the proton-proton collision axis ($R_{xy}$) of at least 0.4 mm. The vertex is required to have at least five tracks reconstructed in the LHCb vertex detector. The analysis is sensitive to $\pi _{\text {v}}$ particles with a mass between 25 and 50$\,\mathrm{GeV}/c^{2}$ and a lifetime between 2 and 500 ps. The lifetime range is limited due to the presence of large prompt backgrounds at short decay times and the acceptance of the vertex detector for long decay times. The lower boundary on the mass range arises from the requirement to identify two hadronic jets while the upper boundary is driven by the geometric acceptance of the detector.

This paper presents an update of an earlier analysis, which considered only the data set corresponding to an integrated luminosity of 0.62$\,\mathrm{fb}^{-1}$ collected at $\sqrt{s}=7$ TeV [9]. Similar searches for hidden-valley particles decaying to jet pairs were performed by the D0 [10], CDF [11], ATLAS [12–14] and CMS [15] collaborations. Compared to these analyses, this search is sensitive to $\pi _{\text {v}}$ particles with relatively low mass and lifetime. The LHCb collaboration has also performed a search for events with two displaced high-multiplicity vertices [16] and a search for events with a lepton from a high-multiplicity displaced vertex [17] in the context of SUSY models, and several searches for so far unknown long-lived particles in *B*-meson decays [18–21].

## Detector and event simulation

The LHCb detector [22, 23] is a single-arm forward spectrometer covering the pseudorapidity range $2<\eta <5$, designed for the study of particles containing $b$ or $c$ quarks. The detector includes a high-precision tracking system consisting of a silicon-strip vertex detector (VELO) surrounding the *pp* interaction region, a large-area silicon-strip detector located upstream of a dipole magnet with a bending power of about $4{\mathrm {\,Tm}}$, and three stations of silicon-strip detectors and straw drift tubes placed downstream of the magnet. The tracking system provides a measurement of the momentum, $p$, of charged particles with a relative uncertainty that varies from 0.5% at low momentum to 1.0% at 200$\,\mathrm{GeV}/c$. The minimum distance of a track to a primary vertex (PV), the impact parameter (IP), is measured with a resolution of $(15+({29}{\,\mathrm{GeV}/c})/p_\mathrm{T})\;{\mu \mathrm{m}}$, where $p_\mathrm{T}$ is the component of the momentum transverse to the collision axis. Different types of charged hadrons are distinguished using information from two ring-imaging Cherenkov detectors. Photons, electrons and hadrons are identified by a calorimeter system consisting of scintillating-pad (SPD) and preshower detectors, an electromagnetic calorimeter and a hadronic calorimeter. Muons are identified by a system composed of alternating layers of iron and multiwire proportional chambers.

The model for the production of $\pi _{\text {v}}$ particles through the Higgs portal is fully specified by three parameters: the mass of the Higgs boson and the mass and lifetime of the $\pi _{\text {v}}$. The Higgs boson mass is taken to be 125$\,\mathrm{GeV}/c^{2}$, and its production through the gluon-gluon fusion process is simulated with the Pythia8 generator [24], with a specific LHCb configuration [25] and using the CTEQ6 leading-order set of parton density functions [26]. The interaction of the generated particles with the detector, and its response, are implemented using the Geant4 toolkit [27, 28] as described in Ref. [29]. Signal samples with $\pi _{\text {v}}$ masses of 25, 35, 43 and 50$\,\mathrm{GeV}/c^{2}$ and lifetimes of 10 and 100 ps are generated. In the simulated events the long-lived particles decay exclusively as $\pi _{\text {v}}\rightarrow {b\bar{b}}$, since this decay mode is generally preferred in the Higgs portal model. Samples with decays to $c$- and $s$-quark pairs are generated as well, but only in the scenario with a mass of 35$\,\mathrm{GeV}/c^{2}$ and a lifetime of 10 ps.

## Event selection

The experimental signature for this analysis is a single displaced vertex with two associated jets. Only decays that produce a sufficient number of tracks in the VELO for a vertex to be reconstructed are considered. Due to the geometry of the vertex detector, this restricts the sample to decay points up to about 200 mm from the nominal interaction point along the beam direction, and up to about 30 mm in the transverse direction, thereby limiting the decay time acceptance. The selection strategy is the same as used in the analysis of Ref. [9]. Reconstructed tracks are used to find the decay vertex, and jets are built out of reconstructed particles compatible with originating from that vertex. Constraints on the signal yield are determined from a fit to the dijet invariant mass distribution. The main source of background is displaced vertices from heavy-flavour decays or interactions of particles with detector material. To take into account the strong dependence of the background level on the separation from the beam axis, different selection criteria are used in different bins of $R_{xy}$, and the final fit is performed in bins of this variable.

The selection consists of online (trigger) and offline parts. The trigger [30] is divided into a hardware (L0) and a software (HLT) stage. The L0 requires a muon with high $p_\mathrm{T}$ or a hadron, photon or electron with high transverse energy in the calorimeters. In order to reduce the processing time of the subsequent trigger stages, events with a large hit multiplicity in the SPD are discarded. The software stage is divided into two parts, which for this analysis differ between the 2011 and 2012 data. In the 2011 sample, the first software stage (HLT1) requires a single high-$p_\mathrm{T}$ track with a large impact parameter. The HLT1 selection for the 2012 sample was complemented with a two-track vertex signature with looser track quality criteria, in order to improve the efficiency at large displacements. At the second stage of the software trigger (HLT2), events are required to pass either a dedicated inclusive displaced-vertex selection or a standard topological *B* decay selection, which requires a two-, three- or four-track vertex with a significant displacement from all PVs [30]. The inclusive displaced-vertex selection uses an algorithm similar to that used for the LHCb primary vertex reconstruction [31]. A combination of requirements on the minimum number of tracks in the vertex (at least four), the distance $R_{xy}$ of the vertex to the beam axis (at least 0.4 mm), the invariant mass of the particles associated with the vertex (at least 2$\,\mathrm{GeV}/c^{2}$) and the scalar sum $p_\mathrm{T}$ of the tracks that form the vertex (at least 3$\,\mathrm{GeV}/c$), is used to define a set of trigger selections with sufficiently low rate.

Before the offline selection can be applied, the displaced vertex corresponding to the decay of the $\pi _{\text {v}}$ candidate must be reconstructed. For those events in which the HLT2 inclusive displaced-vertex selection was successful, the same vertex candidate found in the trigger is used; this approach differs from that used in the previous LHCb analysis [9] and simplifies the evaluation of systematic uncertainties. For events selected only by the topological *B* trigger, a modified version of the algorithm is run on the output of the offline reconstruction with the following criteria: vertices with $0.4< R_{xy}{} < {1}\, \mathrm{mm}$ must have at least eight tracks and the invariant mass of the system must exceed 10$\,\mathrm{GeV}/c^{2}$, vertices with $1< R_{xy}{} < {5}\, \mathrm{mm}$ must have at least six tracks, and those with $R_{xy}{} > {5}\, \mathrm{mm}$ must have at least five tracks. To exclude background due to interactions with the detector material, vertices inside a veto region around the VELO detector elements are discarded. Events with many parallel displaced tracks, which can arise from machine background, are identified by the azimuthal distribution of hits in the VELO and are also discarded.

Next, jets are reconstructed following a particle flow approach. The same set of inputs as in Ref. [32] is used, namely tracks of charged particles and calorimeter energy deposits, after subtraction of the energy associated with charged particles. To remove background, tracks that are compatible with coming from a PV, tracks with a smaller impact parameter to any primary vertex than to the displaced vertex, and tracks that have an impact parameter to the displaced vertex larger than 2 mm are all discarded. The anti-$k_T$ jet clustering algorithm is used [33], with a distance parameter of $R=0.7$. The jet momentum and jet mass are calculated from the four-vectors of all constituents of the jet. In simulated events the jet energy response is found to be close to unity except for the lowest jet momenta, near the minimally required transverse momentum of ${5}{\,\mathrm{GeV}/c}$. Therefore, no jet energy correction was applied for this search.

To enhance the jet purity the fraction of the jet energy carried by charged particles should be at least 0.1, there should be at least one track with transverse momentum above 0.9$\,\mathrm{GeV}/c$, no pair of constituents should carry 90% of the jet energy, and no single charged or neutral constituent should contribute more than 70 or 50% of the total energy, respectively. To ensure that they can reliably be associated to a vertex, the jets are also required to have at least two constituents with track segments in the VELO. To account for differences in trigger and background conditions, for the 2012 data this requirement was tightened to at least four segments for $R_{xy}< {1}\, \mathrm{mm}$, and at least three segments for $1< R_{xy}< {2}\, \mathrm{mm}$. For each jet an origin point is reconstructed from the jet constituents with VELO information. The jet trajectory is defined based on this origin point and the momentum of the jet. Any jet whose trajectory does not point back to the candidate vertex within 2 mm, or points more closely to a primary vertex, is removed. Only candidates with at least two jets passing these criteria are retained.

Two final criteria are applied to the dijet candidates. The first is that the momentum vector of the dijet candidate should be aligned with the displacement vector from a PV to the reconstructed vertex position. This is implemented as a requirement on the dijet invariant mass divided by the corrected mass, $m/m_\text {corr} > 0.7$. The corrected mass is computed as $m_\text {corr} = \sqrt{m^2+(p\sin \theta )^2} + p\sin \theta$ [34], where $m$ and $p$ are the reconstructed mass and momentum of the dijet, and $\theta$ is the minimum angle between the momentum vector and the displacement vectors to the vertex from any PV in the event. A requirement on $m/m_\text {corr}$ is preferred over one on the angle $\theta$ itself, since its efficiency depends less strongly on the boost and the mass of the candidate [35]. The second criterion is that the kinematic separation of the jets should satisfy $\Delta R = \sqrt{(\Delta \eta )^2+(\Delta \phi )^2} < 2.2$, where $\Delta \eta$ and $\Delta \phi$ are the pseudorapidity and azimuthal angle differences between the two jets, respectively. This reduces the tail in the dijet invariant mass distribution by suppressing the remaining back-to-back dijet background.

The overall efficiency to reconstruct and select displaced $\pi _{\text {v}}$ decays in the simulated samples is summarized in Table 1 for the 2011 and 2012 data taking conditions. A large part of the inefficiency is due to the detector acceptance, which is about 13% (8%) and 6.5% (5.5%) for $\pi _{\text {v}}$ particles with a lifetime of 10 ps (100 ps) and masses of 25 and 50$\,\mathrm{GeV}/c^{2}$, respectively. Other important contributions are due to the selection on the displacement from the beamline, requirements on the minimum number of tracks forming the vertex, the material interaction veto, the reduction in VELO tracking efficiency at large displacements, and the jet selection [36]. The efficiency for long-lived particles decaying to $s$- and $c$-quark pairs is higher than for decays to $b$-quark pairs due to the larger number of tracks originating directly from the $\pi _{\text {v}}$ decay vertex.

**Table 1:** Number of selected candidates per generated $H^{0} \rightarrow \pi _{\text {v}}\pi _{\text {v}}$ event (efficiency) in percent for different ${\pi _{\text {v}}} \rightarrow {{q}{}{\overline{q}}{}}$, ${q}=b, c, s$ models for 2011 and 2012 data taking conditions, as derived from simulation. The relative statistical uncertainty on the efficiency due to the limited size of the simulated sample is less than a few percent

	$\pi _{\text {v}}$ mass ($\mathrm{GeV}/c^{2}$)	2011		2012	
		10 ps	100 ps	10 ps	100 ps
${\pi _{\text {v}}}\rightarrow {{b\bar{b}}{}}$	25	0.45	0.097	0.46	0.111
${\pi _{\text {v}}}\rightarrow {{b\bar{b}}{}}$	35	0.80	0.176	0.83	0.224
${\pi _{\text {v}}}\rightarrow {{b\bar{b}}{}}$	43	0.73	0.190	0.77	0.222
${\pi _{\text {v}}}\rightarrow {{b\bar{b}}{}}$	50	0.49	0.141	0.54	0.171
${\pi _{\text {v}}}\rightarrow {{c\bar{c}}{}}$	35	1.35		1.35	
${\pi _{\text {v}}}\rightarrow {{s\bar{s}}{}}$	35	1.30		1.19	

## Systematic uncertainties

Systematic uncertainties on the efficiency are obtained from studies of data-simulation differences in control samples. They are reported in Tables 2 and 3, for the 2011 and 2012 conditions, respectively, and discussed in more detail below. Uncertainties on the signal efficiency due to parton-density distributions, the simulation of fragmentation and hadronization, and the Higgs boson production cross-section and kinematics are not taken into account.

**Table 2:** Overview of the contributions to the relative systematic uncertainty on the signal efficiency and luminosity (in percent) for different signal samples in 2011 conditions. The uncertainty on the total efficiency is obtained by summing the individual contributions in quadrature

$\pi _{\text {v}}$ mass ($\,\mathrm{GeV}/c^{2}$)	25		35		43		50		35, $c\bar{c}$	35, $s\bar{s}$
$\pi _{\text {v}}$ lifetime (ps)	10	100	10	100	10	100	10	100	10	10
Tracking efficiency	4.2	4.1	3.3	3.2	3.0	2.8	3.0	2.7	1.8	1.7
Vertex finding	3.8	4.2	3.3	3.9	2.8	3.7	3.7	2.6	2.9	2.8
Jet reconstruction	3.1	3.1	1.6	1.6	0.7	0.7	0.5	0.5	0.9	1.0
Jet identification	3.0	3.0	3.0	3.0	3.0	3.0	3.0	3.0	3.0	3.0
Jet direction	7.0	7.0	6.0	6.0	7.4	7.4	8.5	8.5	5.9	5.7
L0	4.0	4.0	3.0	3.0	3.0	3.0	2.0	2.0	1.8	2.1
$N_{\text {SPD}}$	1.7	1.7	2.0	2.0	1.6	1.6	2.3	2.3	1.7	1.6
HLT1	2.0	2.0	2.0	2.0	2.0	2.0	2.0	2.0	2.0	2.0
HLT2	3.0	3.0	3.0	3.0	3.0	3.0	3.0	3.0	3.0	3.0
Total efficiency	11.5	11.6	9.8	10.0	10.3	10.5	11.2	10.9	8.7	8.6
Luminosity	1.7	1.7	1.7	1.7	1.7	1.7	1.7	1.7	1.7	1.7

**Table 3:** Overview of the contributions to the relative systematic uncertainty on the signal efficiency and luminosity (in percent) for different signal samples in 2012 conditions. The uncertainty on the total efficiency is obtained by summing the individual contributions in quadrature

$\pi _{\text {v}}$ mass ($\,\mathrm{GeV}/c^{2}$)	25		35		43		50		35, $c\bar{c}$	35, $s\bar{s}$
$\pi _{\text {v}}$ lifetime (ps)	10	100	10	100	10	100	10	100	10	10
Tracking efficiency	3.1	2.8	2.4	2.4	2.2	2.1	2.0	1.7	1.2	1.1
Vertex finding	4.2	4.5	3.8	4.4	3.4	4.1	3.1	3.9	3.4	3.5
Jet reconstruction	2.7	2.7	1.1	1.1	0.7	0.7	0.3	0.3	0.9	1.0
Jet identification	3.0	3.0	3.0	3.0	3.0	3.0	3.0	3.0	3.0	3.0
Jet direction	5.8	5.8	5.3	5.3	6.1	6.1	7.9	7.9	5.3	5.8
L0	4.0	4.0	2.5	2.5	2.0	2.0	2.0	2.0	2.0	2.0
$N_{\text {SPD}}$	2.2	2.2	2.5	2.5	2.5	2.5	2.5	2.5	2.4	2.1
HLT1	2.0	2.0	2.0	2.0	2.0	2.0	2.0	2.0	2.0	2.0
HLT2	3.0	3.0	3.0	3.0	3.0	3.0	3.0	3.0	3.0	3.0
Total efficiency	10.5	10.6	9.2	9.4	9.1	9.5	10.4	10.6	8.6	8.9
Luminosity	1.2	1.2	1.2	1.2	1.2	1.2	1.2	1.2	1.2	1.2

The vertex reconstruction efficiency can be split into two parts, namely the track reconstruction efficiency and the vertex finding efficiency. The track reconstruction efficiency is described by the simulation to within a few percent, including for highly displaced and low-momentum tracks [37–39]. The effect of a systematic change in this efficiency is studied by randomly removing 2% of the signal tracks and reapplying all selection criteria.

The vertex finding algorithm is not fully efficient even if all tracks are reconstructed. In particular, the efficiency to find a low-multiplicity secondary vertex is reduced in the proximity of a high-multiplicity PV. The effect is studied in data and simulation using exclusively reconstructed $B^{0}\rightarrow J/\varPsi K^{*0}$ decays, which can be selected with high purity without tight requirements on the vertex. The efficiency for the displaced vertex reconstruction algorithm to find the $B^0$ candidate is measured as a function of the displacement $R_{xy}$ in data and simulation [36]. The difference, weighted by the $R_{xy}$ distribution of the signal candidates, is used to derive a systematic uncertainty.

Systematic uncertainties related to the jet reconstruction can be introduced in two ways: through differences between data and simulation in the jet reconstruction efficiency and through differences between data and simulation in the resolution on the jet energy and direction, which enter the dijet candidate kinematic and $m/m_\text {corr}$ selection and the dijet invariant mass shape. The jet reconstruction efficiency has been studied previously in measurements of the ${Z}+\text {jet}$ and ${Z}+b\text {-jet}$ cross-sections and was found to be consistent between data and simulation [32, 40]. The ${{Z}} \rightarrow {{\mu ^{+}\mu ^{-}}}+\text {jet}$ sample is used to study jet-related systematic effects for this analysis as well. To mimic the selection of the particle-flow inputs, the PV associated to the ${Z}$ is used as a proxy for the displaced vertex.

**Fig. 1:**
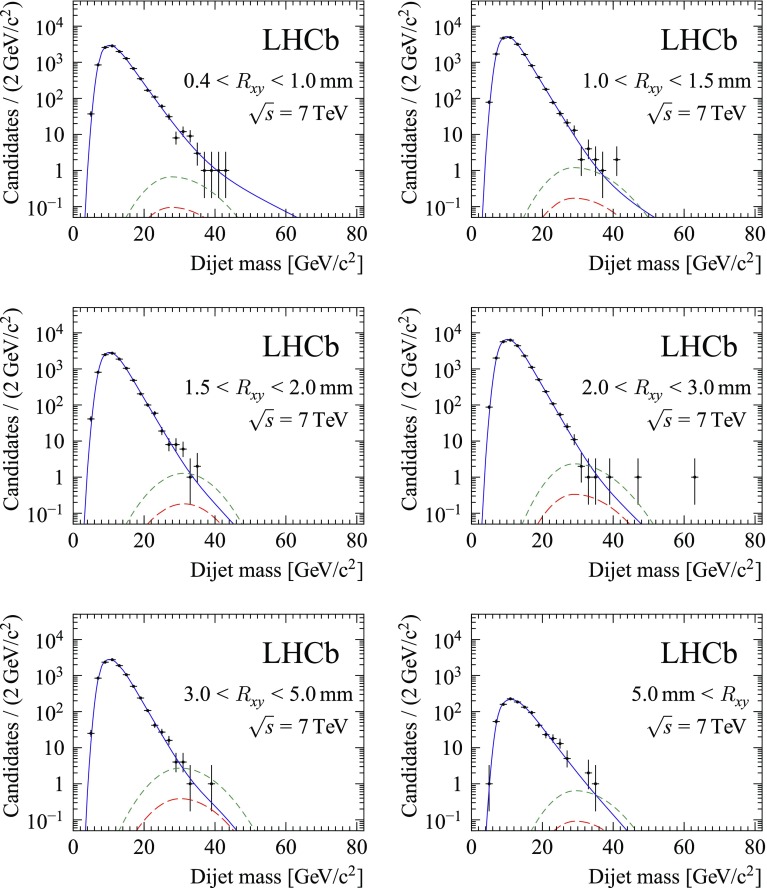
Dijet invariant mass distribution in the different $R_{xy}$ bins, for the 2011 data sample. For illustration, the best fit with a signal $\pi _{\text {v}}$ model with mass 35$\,\mathrm{GeV}/c^{2}$ and lifetime 10 ps is overlaid. The solid blue line indicates the total background model, the short-dashed green line indicates the signal model for signal strength $\mu =1$, and the long-dashed red line indicates the best-fit signal strength

**Fig. 2:**
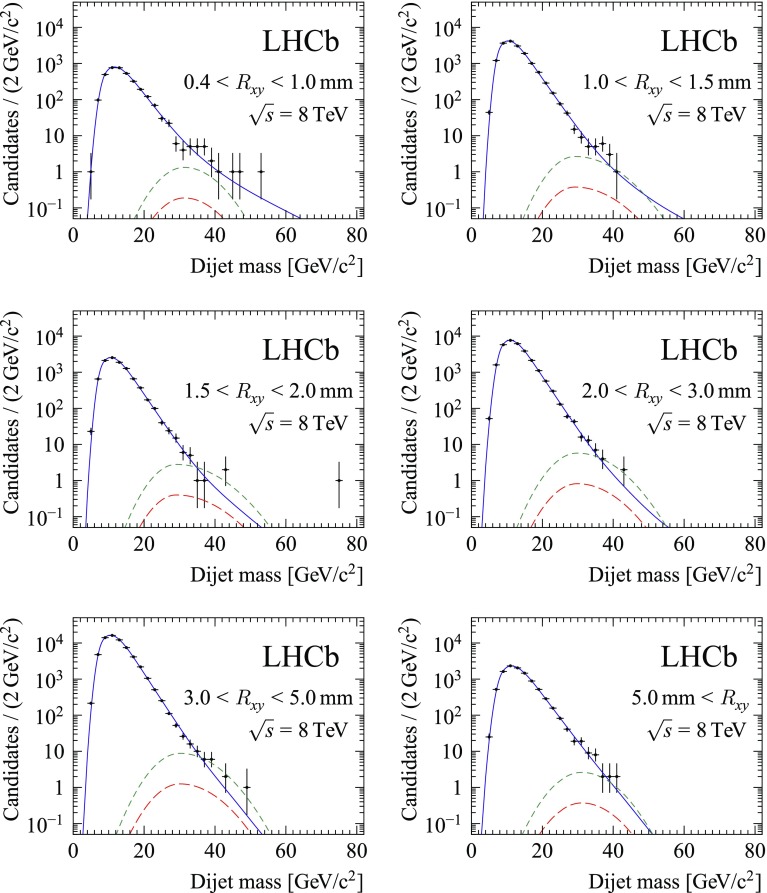
Dijet invariant mass distribution in the different $R_{xy}$ bins, for the 2012 data sample. For illustration, the best fit with a signal $\pi _{\text {v}}$ model with mass 35$\,\mathrm{GeV}/c^{2}$ and lifetime 10 ps is overlaid. The solid blue line indicates the total background model, the short-dashed green line indicates the signal model for signal strength $\mu =1$, and the long-dashed red line indicates the best-fit signal strength

**Fig. 3:**
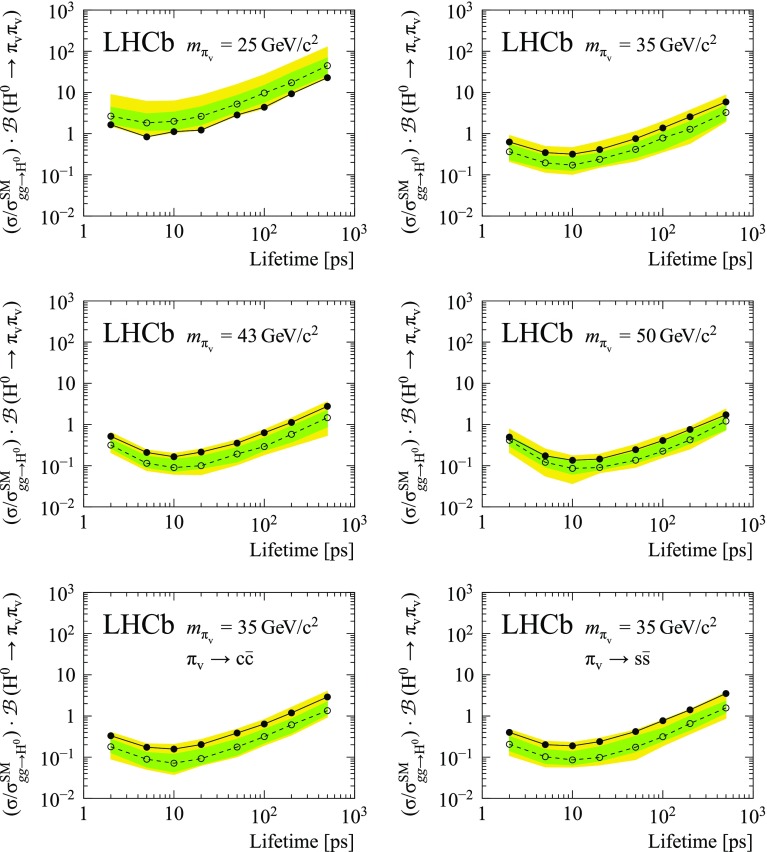
Expected (open circles and dotted line) and observed (filled circles and solid line) upper limit versus lifetime for different $\pi _{\text {v}}$ masses and decay modes. The green (dark) and yellow (light) bands indicate the quantiles of the expected upper limit corresponding to $\pm 1\sigma$ and $\pm 2\sigma$ for a Gaussian distribution. The decay ${\pi _{\text {v}}{}}\rightarrow {{b\bar{b}}{}}$ is assumed, unless specified otherwise

The difference between data and simulation with the largest impact on the jet reconstruction efficiency is the energy response to low-$p_\mathrm{T}$ jets, close to the threshold of 5$\,\mathrm{GeV}/c$. The sensitivity to a different energy response in data and simulation is evaluated by increasing the minimum jet $p_\mathrm{T}$ for candidates passing the full offline selection by 10%, which is the uncertainty on the jet energy scale. The change in the overall selection efficiency is assigned as a systematic uncertainty. By replacing the jet identification criteria with a requirement on the $p_\mathrm{T}$ balance between the leading jet and the $Z$ boson, the ${Z}\rightarrow {\mu ^{+}\mu ^{-}}$ sample can also be used to study the difference in jet identification efficiency between data and simulation. No difference larger than 3% relative is seen, which is assigned as a systematic uncertainty.

To validate the simulation of the jet-direction resolution the jet-direction is estimated separately with the charged and neutral components of the jet in ${Z}{}+\text {jet}$ events. The distribution of the charged-neutral difference in the estimated direction is found to be consistent between data and simulation for both the $\eta$ and the $\phi$ projection, and across the full range of $p_\mathrm{T}$. To quantify the effect on the $\pi _{\text {v}}$ signal efficiency, an additional smearing to the jet-direction is applied to jets of selected candidates in the simulation. The jet angles with respect to the beam direction are smeared independently in the horizontal and vertical planes by about one third of the resolution, which is the largest value compatible with the comparison of data and simulation in ${Z}{}+\text {jet}$ events.

The systematic uncertainty related to the L0 trigger selection consists of two parts, due to differences in the L0 calorimeter trigger response between data and simulation, and due to the difference between data and simulation in the distribution of the SPD hit multiplicity $N_\text {SPD}$. The first is evaluated by studying the L0 calorimeter trigger response on jets reconstructed in ${Z}{}+\text {jet}$ events, where the trigger decision is made based on the ${Z}\rightarrow {\mu ^{+}\mu ^{-}}$ decay products, and is independent of the jet. The observed data-simulation differences are propagated to the $\pi _{\text {v}}$ reconstruction efficiency and correspond to systematic uncertainties of 2–4%, depending on the $\pi _{\text {v}}$ mass. Jets in ${Z}{}+\text {jet}$ events are mostly light-quark jets, while our benchmark signal decays to *b* quarks. It is found in simulated events that the efficiency of the L0 calorimeter trigger is practically independent of jet flavour. A small fraction of *b*-quark jets is triggered exclusively by the L0 muon trigger, which is well modelled in the simulation.

The second part of the L0 systematic uncertainty arises because the SPD multiplicity is not well described in the simulation. This effect is studied with a ${Z}\rightarrow {\mu ^{+}\mu ^{-}}$ sample triggered by the dimuon L0 selection, which applies only a loose selection on this quantity. An efficiency correction is derived, which is about 90% for 2011 data, and about 85% for 2012 data, with an uncertainty of 2–3%. The difference in the correction between the different $\pi _{\text {v}}$ models is smaller than the systematic variation. This correction is applied to the overall detection efficiency derived from the simulation and the uncertainty is taken as a systematic uncertainty.

The differences between data and simulation in the HLT1 selection are dominated by the track reconstruction efficiency, which was discussed above, and additional track quality criteria. One such difference is due to a requirement on the number of VELO hits for displaced tracks. It is characterized using $B^{0}\rightarrow J/\varPsi K^{*0}$ decays selected with triggers that do not apply such a requirement. For this sample the selection efficiency was found to be 2% higher in data than in simulated events, which is assigned as a systematic uncertainty. For $\pi _{\text {v}}$ decays the final-state track multiplicity is larger, which dilutes effects due to a mismodelling of the single-track efficiency.

The main source of systematic uncertainty in the HLT2 selection is the vertex reconstruction efficiency, which was discussed above. The efficiency of the topological $B$ trigger, which is relevant for a subset of the candidates, is accurately described in simulation. It is measured as a function of $R_{xy}$ in data and simulation using $B^{0}\rightarrow J/\varPsi K^{*0}$ candidates that are selected by a different, dimuon-based, trigger criterion. A maximum difference of 2–3% is observed, which is assigned as a systematic uncertainty.

## Results

Constraints on the presence of a signal are derived from a fit to the dijet invariant mass distributions, shown in Figs. 1 and 2. To take advantage of the difference in the $R_{xy}$ distribution for background and signal, the data are divided into six $R_{xy}$ bins. The data are further split according to data taking year to account for differences in running conditions and Higgs boson production cross-section. The signal efficiency for each $R_{xy}$ bin is obtained from the simulated samples with $\pi _{\text {v}}$ lifetimes of 10 and 100 ps, with the decay time distributions reweighted to mimic other lifetime hypotheses as needed.

Results are presented as upper limits on the signal strength $\mu \equiv (\sigma /\sigma _{gg\rightarrow {H^0}{}}^{SM})\cdot \mathcal {B}({H^{0} \rightarrow \pi _{\text {v}}\pi _{\text {v}}}{})$, where $\sigma$ is the excluded signal cross-section, $\sigma _{gg\rightarrow {H^0}{}}^{SM}$ is the SM Higgs boson production cross-section via the gluon fusion process and $\mathcal {B}({H^{0} \rightarrow \pi _{\text {v}}\pi _{\text {v}}}{})$ is the branching fraction of the Higgs boson decay to $\pi _{\text {v}}{}$ particles. The branching fraction $\mathcal {B}_{{q}{\overline{q}}}$ of the $\pi _{\text {v}}$ particle to the $q\bar{q}$ final state (with ${q\bar{q}}={b\bar{b}}$, ${c\bar{c}}$ or ${s\bar{s}}$ depending on the final state under study) is assumed to be 100%. If the decay width of the $\pi _{\text {v}}$ particle is dominated by other decays than that under study, the limits scale as $1/(\mathcal {B}_{{q}{}{\overline{q}}{}}(2-\mathcal {B}_{{q}{}{\overline{q}}{}}))$. The Higgs boson production cross-section is assumed to be 15.11 pb at 7 TeV and 19.24 pb at 8 TeV [41].

The $\text {CL}_s$ method [42] is used to determine upper limits. The profile likelihood ratio $q_\text {PLL}^\mu = {L(\mu ,\hat{\theta }(\mu ))}/{L(\hat{\mu },\hat{\theta })}$ is chosen as a test statistic, where $L(\mu ,\theta )$ denotes the likelihood as a function of $\mu$ and a set of nuisance parameters $\theta$, which are also extracted from the data; $L(\mu ,\hat{\theta }(\mu ))$ is the maximum likelihood for a hypothesized value of $\mu$ and $L(\hat{\mu },\hat{\theta })$ is the global maximum likelihood. To estimate the sensitivity of the analysis and the significance of a potential signal, the expected upper limit quantiles in the case of zero signal are also evaluated.

**Table 4:** Observed 95% CL signal strength ($\mu$) upper limits for different $\pi _{\text {v}}$ models

$\pi _{\text {v}}$ mass	$\pi _{\text {v}}$ lifetime (ps)							
	2	5	10	20	50	100	200	500
25$\,\mathrm{GeV}/c^{2}$	1.64	0.83	1.12	1.22	2.84	4.37	9.28	22.82
35$\,\mathrm{GeV}/c^{2}$	0.63	0.35	0.32	0.41	0.76	1.37	2.56	5.86
43$\,\mathrm{GeV}/c^{2}$	0.52	0.21	0.16	0.21	0.35	0.63	1.12	2.77
50$\,\mathrm{GeV}/c^{2}$	0.50	0.17	0.14	0.15	0.25	0.41	0.76	1.72
35$\,\mathrm{GeV}/c^{2}$, $\pi _{\text {v}}{}\rightarrow c{}\bar{c{}}$	0.33	0.17	0.16	0.20	0.39	0.64	1.19	2.90
35$\,\mathrm{GeV}/c^{2}$, $\pi _{\text {v}}{}\rightarrow s{}\bar{s{}}$	0.40	0.20	0.19	0.24	0.42	0.77	1.41	3.51

For each value of $\mu$ and $\theta$ the likelihood is evaluated as $L(\mu ,\theta ) = \prod _{i} P(x_i;\mu ,\theta )$, where *P* is the probability density for event *i* and the product runs over all selected events. The observables $x_i$ for each candidate include the dijet mass, $R_{xy}$ bin and data taking year. For each $R_{xy}$ bin and data taking year, the invariant mass distribution is modelled by the sum of background and signal components. The distribution for the signal is modelled as a Gaussian distribution whose parameters are obtained from fully simulated signal events. For the background distribution an empirical model, outlined below, is adopted.

Background candidates can be categorized into two contributions. The first category is mostly due to the combination of a heavy-flavour decay vertex or an interaction with detector material with particles from a primary interaction. This contribution has a steeply decreasing invariant mass spectrum. Following the approach in Ref. [9], the distribution is modelled by the convolution of a falling exponential distribution with a bifurcated Gaussian. All parameters of this background model are free to vary in the fit.

The second category is due to Standard Model dijet events. These events have candidates with jets that are approximately back-to-back in the transverse plane. It is suppressed by the selection on the dijet opening angle $\Delta R$. Its remaining contribution has a less steeply falling mass spectrum. It is described in the fit with a similar functional shape as for the first category, but with the parameters and the relative yields in the different bins fixed from a fit to the invariant mass distribution of candidates that fail the $\Delta R$ requirement. In the final fit only the total normalization of this component is varied. The second component is new compared to the model used for the previous analysis [9]. It leads to a better description of the high-mass tail, at the expense of one extra fit parameter for each data taking year. It was found that the result of the fit is not sensitive to the exact $\Delta R$ requirement used to select the events for this component.

All parameters of the fit to the invariant mass distribution are allowed to float independently in each bin, except for the following nuisance parameters: the dijet invariant mass scale, the overall signal efficiency, and the normalization for the second background contribution. All relevant systematic uncertainties are incorporated in the fit model: the overall uncertainty on the efficiency, as described in Sect. 4, the uncertainty on the dijet invariant mass scale, and the uncertainties on the shape parameters and relative normalisation arising from the finite size of the simulated signal samples. Gaussian constraints on these parameters are added to the likelihood.

Alternatives have been considered for the background mass model, in particular with an additional less steeply falling exponential to describe the tail. With these models the estimated background yield at higher mass is similar or larger than with the nominal background model, leading to tighter limits on the signal. As the nominal model gives the most conservative limit, no additional systematic uncertainty is assigned for background modeling.

**Fig. 4:**
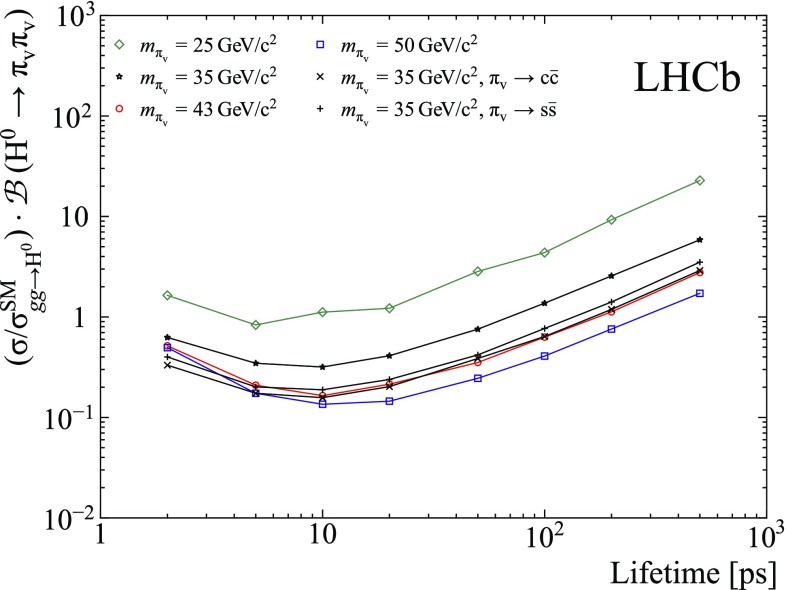
Observed upper limit versus lifetime for different $\pi _{\text {v}}$ masses and decay modes. The decay ${\pi _{\text {v}}{}}\rightarrow {{b\bar{b}}{}}$ is assumed, unless specified otherwise

There is no significant excess of signal in the data. Upper limits at 95% confidence level (CL) as a function of lifetime for hidden-valley models with different $\pi _{\text {v}}$ mass and decay mode are shown in Fig. 3 and summarized in Table 4 and Fig. 4. The best sensitivity is obtained for a mass of about 50$\,\mathrm{GeV}/c^{2}$ and a lifetime of about 10 ps. The main improvements with respect to the previous result [9] are due to the enlarged data sample, the improved trigger selections, and the addition of the $R_{xy}$ bin above 5 mm, which contributes to the increased sensitivity at larger lifetimes.

## Conclusion

Results have been presented from a search for long-lived particles with a mass in the range 25–50$\,\mathrm{GeV}/c^{2}$ and a lifetime between 2 and 500 ps. The particles are assumed to be pair-produced in the decay of a 125$\,\mathrm{GeV}/c^{2}$ Standard-Model-like Higgs boson and to decay into two jets. Besides decays to $b\bar{b}$, which are the best motivated in the context of hidden-valley models [1, 2], also decays to $c\bar{c}$ and $s\bar{s}$ quark pairs are considered. No evidence for so far unknown long-lived particles is observed and limits are set as a function of mass and lifetime. These measurements complement other constraints on this production model at the LHC [13, 15] by placing stronger constraints at small masses and lifetimes.

## References

[1] Strassler MJ, Zurek KM (2007). Echoes of a hidden valley at hadron colliders. Phys. Lett. B.

[2] Strassler MJ, Zurek KM (2008). Discovering the Higgs through highly-displaced vertices. Phys. Lett. B.

[3] Chang S, Dermisek R, Gunion JF, Weiner N (2008). Nonstandard Higgs boson decays. Annu. Rev. Nucl. Part. Sci..

[4] Craig N, Katz A, Strassler M, Sundrum R (2015). Naturalness in the dark at the LHC. JHEP.

[5] Curtin D, Verhaaren CB (2015). Discovering uncolored naturalness in exotic Higgs decays. JHEP.

[6] ATLAS collaboration, G. Aad et al., Observation of a new particle in the search for the Standard Model Higgs boson with the ATLAS detector at the LHC. Phys. Lett. B **716**, 1 (2012). arXiv:1207.7214

[7] CMS collaboration, S. Chatrchyan et al., Observation of a new boson at a mass of 125 GeV with the CMS experiment at the LHC. Phys. Lett. B **716**, 30 (2012). arXiv:1207.7235

[8] ATLAS and CMS collaborations, G. Aad et al., Measurements of the Higgs boson production and decay rates and constraints on its couplings from a combined ATLAS and CMS analysis of the LHC pp collision data at $$\sqrt{s}=7$$ and 8 TeV. JHEP **08**, 045 (2016). arXiv:1606.02266

[9] LHCb collaboration, R. Aaij et al., Search for long-lived particles decaying to jet pairs. Eur. Phys. J. C **75**, 152 (2015). arXiv:1412.3021 10.1140/epjc/s10052-015-3344-6 PMC4423877 25983649

[10] D0 collaboration, V.M. Abazov et al., Search for resonant pair production of long-lived particles decaying to $$b\bar{b}$$ in $$p\bar{p}$$ collisions at $$\sqrt{s}= 1.96$$ TeV. Phys. Rev. Lett. **103**, 071801 (2009). arXiv:0906.1787 10.1103/PhysRevLett.103.071801 19792632

[11] CDF collaboration, T. Aaltonen et al., Search for heavy metastable particles decaying to jet pairs in pp collisions at $$\sqrt{s}= 1.96$$ TeV. Phys. Rev. D **85**, 012007 (2012). arXiv:1109.3136

[12] ATLAS collaboration, G. Aad et al., Search for a light Higgs boson decaying to long-lived weakly-interacting particles in proton–proton collisions at $$\sqrt{s}=7$$ TeV with the ATLAS detector. Phys. Rev. Lett. **108**, 251801 (2012). arXiv:1203.1303 10.1103/PhysRevLett.108.251801 23004585

[13] ATLAS collaboration, G. Aad et al., Search for long-lived, weakly interacting particles that decay to displaced hadronic jets in proton–proton collisions at $$\sqrt{s}= 8$$ TeV with the ATLAS detector. Phys. Rev. D **92**, 012010 (2015). arXiv:1504.03634

[14] ATLAS collaboration, G. Aad et al., Search for pair-produced long-lived neutral particles decaying in the ATLAS hadronic calorimeter in pp collisions at $$\sqrt{s}= 8$$ TeV. Phys. Lett. B **743**, 15 (2015). arXiv:1501.04020

[15] CMS collaboration, V. Khachatryan et al., Search for long-lived neutral particles decaying to quark–antiquark pairs in proton–proton collisions at $$\sqrt{s}= 8$$ TeV. Phys. Rev. D **91**, 012007 (2015). arXiv:1411.6530

[16] LHCb collaboration, R. Aaij et al., Search for Higgs-like bosons decaying into long-lived exotic particles. Eur. Phys. J. C **76**, 664 (2016). arXiv:1609.03124 10.1140/epjc/s10052-016-4489-7 PMC5335594 28316499

[17] LHCb collaboration, R. Aaij et al., Search for massive long-lived particles decaying semileptonically in the LHCb detector. Eur. Phys. J. C **77**, 224 (2017). arXiv:1612.00945 10.1140/epjc/s10052-017-4744-6 PMC5408995 28515664

[18] LHCb collaboration, R. Aaij et al., Search for Majorana neutrinos in $$B^{-} \rightarrow \pi ^{+}\mu ^{-}\mu ^{-}$$ decays. Phys. Rev. Lett. **112**, 131802 (2014). arXiv:1401.5361 10.1103/PhysRevLett.112.131802 24745405

[19] LHCb collaboration, R. Aaij et al., Searches for Majorana neutrinos in $$B^{-}$$ decays. Phys. Rev. D **85**, 112004 (2012). arXiv:1201.5600

[20] LHCb collaboration, R. Aaij et al., Search for hidden-sector bosons in $$B^{0}\rightarrow K^{*0}\chi (\mu ^{+}\mu ^{-})$$ decays. Phys. Rev. Lett. **115**, 161802 (2015). arXiv:1508.04094 10.1103/PhysRevLett.115.161802 26550866

[21] LHCb collaboration, R. Aaij et al., Search for long-lived scalar particles in $$B^{+}\rightarrow K^{+}\chi (\mu ^{+}\mu ^{-})$$ decay. Phys. Rev. D **95**, 071101 (2017). arXiv:1612.07818

[22] LHCb collaboration, A.A. Alves Jr. et al., The LHCb detector at the LHC. JINST **3**, S08005 (2008)

[23] LHCb collaboration, R. Aaij et al., LHCb detector performance. Int. J. Mod. Phys. A **30**, 1530022 (2015). arXiv:1412.6352

[24] Sjöstrand T, Mrenna S, Skands P (2008). A brief introduction to PYTHIA 8.1. Comput. Phys. Commun..

[25] Belyaev I (2011). Handling of the generation of primary events in Gauss, the LHCb simulation framework. J. Phys. Conf. Ser..

[26] Pumplin J (2002). New generation of parton distributions with uncertainties from global QCD analysis. JHEP.

[27] Geant4 collaboration, J. Allison et al., Geant4 developments and applications. IEEE Trans. Nucl. Sci. **53**, 270 (2006)

[28] Geant4 collaboration, S. Agostinelli et al., Geant4: a simulation toolkit. Nucl. Instrum. Methods A **506**, 250 (2003)

[29] Clemencic M (2011). The LHCb simulation application, Gauss: design, evolution and experience. J. Phys. Conf. Ser..

[30] Aaij R (2013). The LHCb trigger and its performance in 2011. JINST.

[31] M. Kucharczyk, P. Morawski, M. Witek, Primary vertex reconstruction at LHCb. LHCb-PUB-2014-044, Geneva: CERN

[32] LHCb collaboration, R. Aaij et al., Study of forward Z+jet production in pp collisions at $$\sqrt{s}= 7$$ TeV. JHEP **01**, 033 (2014). arXiv:1310.8197

[33] Cacciari M, Salam GP, Soyez G (2008). The anti-$$k_t$$ jet clustering algorithm. JHEP.

[34] SLD collaboration, K. Abe et al., Measurement of $$R_b$$ using a vertex mass tag. Phys. Rev. Lett. **80**, 660 (1998). arXiv:hep-ex/9708015

[35] V. Heijne, Search for long-lived exotic particles at LHCb. PhD thesis (Vrije Universiteit, Amsterdam, 2016), CERN-THESIS-2014-294

[36] P.N.Y. David, Search for exotic long-lived particles with the LHCb detector. PhD thesis (Vrije Universiteit, Amsterdam, 2016), CERN-THESIS-2016-077

[37] LHCb collaboration, R. Aaij et al., Measurement of the track reconstruction efficiency at LHCb. JINST **10**, P02007 (2015). arXiv:1408.1251

[38] LHCb collaboration, R. Aaij et al., Prompt $$K_S^0$$ production in pp collisions at $$\sqrt{s}= 0.9$$ TeV. Phys. Lett. B **693**, 69 (2010). arXiv:1008.3105

[39] LHCb collaboration, R. Aaij et al., Measurement of $$V^{0}$$ production ratios in pp collisions at $$\sqrt{s}= 0.9$$ and 7 TeV. JHEP **08**, 034 (2011). arXiv:1107.0882

[40] LHCb collaboration, R. Aaij et al., Measurement of the Z $$+$$ b-jet cross-section in pp collisions at $$\sqrt{s}= 7$$ TeV in the forward region. JHEP **01**, 064 (2015). arXiv:1411.1264

[41] LHC Higgs Cross Section Working Group, S. Heinemeyer et al., Handbook of LHC Higgs cross sections: 3. Higgs properties. CERN-2013-004. arXiv:1307.1347

[42] Read AL (2002). Presentation of search results: the $$CL_s$$ technique. J. Phys. G.

